# Pilot Lipidomics Study of Copepods: Investigation of Potential Lipid-Based Biomarkers for the Early Detection and Quantification of the Biological Effects of Climate Change on the Oceanic Food Chain

**DOI:** 10.3390/life13122335

**Published:** 2023-12-13

**Authors:** Paul L. Wood, Michael D. Wood, Stan C. Kunigelis

**Affiliations:** 1 Metabolomics Unit, College of Veterinary Medicine, Lincoln Memorial University, 6965 Cumberland Gap Pkwy., Harrogate, TN 37752, USA; 2 Child and Adolescent Psychiatry, BC Children’s and Women’s Hospital & Provincial Health Services Authority, Vancouver, BC V5Z 4H4, Canada; michael.wood@ubc.ca; 3 Imaging and Analysis Center, DeBusk College of Osteopathic Medicine, Lincoln Memorial University, 6965 Cumberland Gap Pkwy., Harrogate, TN 37752, USA; stan.kunigelis@lmunet.edu

**Keywords:** copepods, lipidomics, climate change, oceanic food chain, sentinel species

## Abstract

Maintenance of the health of our oceans is critical for the survival of the oceanic food chain upon which humanity is dependent. Zooplanktonic copepods are among the most numerous multicellular organisms on earth. As the base of the primary consumer food web, they constitute a major biomass in oceans, being an important food source for fish and functioning in the carbon cycle. The potential impact of climate change on copepod populations is an area of intense study. Omics technologies offer the potential to detect early metabolic alterations induced by the stresses of climate change. One such omics approach is lipidomics, which can accurately quantify changes in lipid pools serving structural, signal transduction, and energy roles. We utilized high-resolution mass spectrometry (≤2 ppm mass error) to characterize the lipidome of three different species of copepods in an effort to identify lipid-based biomarkers of copepod health and viability which are more sensitive than observational tools. With the establishment of such a lipid database, we will have an analytical platform useful for prospectively monitoring the lipidome of copepods in a planned long-term five-year ecological study of climate change on this oceanic sentinel species. The copepods examined in this pilot study included a North Atlantic species (*Calanus finmarchicus*) and two species from the Gulf of Mexico, one a filter feeder (*Acartia tonsa*) and one a hunter (*Labidocerca aestiva*). Our findings clearly indicate that the lipidomes of copepod species can vary greatly, supporting the need to obtain a broad snapshot of each unique lipidome in a long-term multigeneration prospective study of climate change. This is critical, since there may well be species-specific responses to the stressors of climate change and co-stressors such as pollution. While lipid nomenclature and biochemistry are extremely complex, it is not essential for all readers interested in climate change to understand all of the various lipid classes presented in this study. The clear message from this research is that we can monitor key copepod lipid families with high accuracy, and therefore potentially monitor lipid families that respond to environmental perturbations evoked by climate change.

## 1. Introduction

Oceans contain the largest ecosystem on our planet, dominated by phytoplankton [1]. Primary producing phytoplankton play an important role in the regulation of global temperature through the seeding of low-lying clouds with volatile sulfur-based organics. In turn, phytoplankton are a major component of the diet of copepods, the dominant zooplankton in the pelagic food chain [2]. As primary consumers, copepods feed heavily on phytoplankton, thereby influencing their role in climate regulation. In this regard, copepods are a keystone species in marine food webs and have been referred to as the singing canaries of the ocean due to their sensitivity to the physiochemical conditions of the oceans [3]. Copepods serve both as a food source for marine fish larvae, including a variety of commercially harvested fish, and they also are critical players in carbon sequestration. In studies of climate change, it has been shown that copepods are intrinsic to the carbon cycle, which involves fixation of atmospheric CO_2_ by photosynthetic algae and the export of this carbon via detrital debris from copepods and other predators to deeper depths of oceans [4,5]. With climate change resulting in elevated oceanic temperatures and pCO_2_ (i.e., ocean acidification), populations of phytoplankton are projected to increase, while those of copepods are projected to decrease [6,7,8,9,10] or shift with regard to dominant copepod species, since *Calanus finmarchicus* (*C. finmarchicus*) is most sensitive to warmer temperatures [11]. Monitoring the numbers of these species is important, but there are limitations to observational methods. It is therefore important to obtain early biomarkers of the viability/health of phyto- and zoo-plankton populations.

“Omics” technologies, which monitor mRNA (transcriptomics), proteins (proteomics), and metabolites (metabolomics), offer a solution to this current problem. Lipidomics is a subfield of metabolomics which provides an in-depth evaluation of lipids involved in structural, signal transduction, and energy functions. High-resolution mass spectrometry (HR-MS) provides a high-capacity workflow which acquires high-precision data that allows for the identification and quantification of a broad range of individual lipid members of a diverse array of lipid families. In the case of our analytical platform, we monitor over 12,000 potential lipids across 181 lipid families. The platform also monitors organic soluble non-lipids from 12 different chemical families. Direct flow infusion analysis (FIA) reduces the risk of sample alteration during processing, reduces the variation introduced by manipulative steps, significantly reduces the sample preparation time, and provides a stable ionization via the supply of a constant lipid concentration during the FIA [12].

In studies of the nutritional quality of phytoplankton, lipidomics evaluations have monitored isoprostanoids, glycerophospholipids (GPL), diacylglycerols (DG), triacylglycerols (TG), monogalactosyl-DG (MGDG), digalactosyl-DG (DGDG), and diacylglycyceryl-trimethylhomoserine (DGTS) lipid families [13,14,15,16,17,18,19,20,21,22]. In addition, tandem mass spectrometry has been used in a number of these studies to define the fatty acid substitutions of lipids. Definition of fatty acid substituents in complex lipids supplies information on the availability and flux of free fatty acids involved in the dynamic and critical processes of lipid remodeling [23]. These processes include altered deacylation/reacylation mechanisms, changes in the utilization of biosynthetic precursors, and altered lipid degradation. Monitoring the fatty acid constituents of complex lipids has a proven track record of utility in the study of responses in the lipidome to environmental changes [24,25,26,27]. It is already established that remodeling of membrane lipids, to maintain membrane fluidity, is a critical process in cellular stability. Work from a number of laboratories has already established that one of the major effects of ocean acidification is to decrease the quality of the lipidome in microalgae [28,29]. The domino effects of this are projected to affect copepods which utilize microalgae as a major nutrient source, and subsequently on fish which feed on copepod populations. Defining these climate-dependent lipid alterations in copepods is our long-term research goal.

The lipidome of *Gammarus fossarum* has been characterized to monitor environmental effects on this freshwater crustacean sentinel species [30]. Lipidomics studies of copepods have been more limited, but have characterized fatty acids and fatty alcohols [31,32,33], wax esters [34,35,36], carotenoids [37,38,39], and copepodamides [40,41,42]. Our goal is to utilize this published data and expand on it to obtain a more detailed picture of the lipidomes of three different copepod species and compare that to the lipidome of a marine alga. With this database, we will next be positioned to move into a longer-term ecological study of the effects of climate change on copepod health and viability. Our data will also provide information on the health of phytoplankton, since these marine organisms represent a major source of nutrition for copepods that are filter feeders. These speculations are based on prior research that has shown large alterations in membrane lipids with environmental stressors on fungi [43], bacteria [44], and algae [45]. Having the first highly quantitative longitudinal data of copepod viability will ultimately be of incredible value to agencies involved in regulatory decision-making.

## 2. Materials and Methods

### 2.1. Copepods

Freeze-dried zooplankton copepods (*Calanus finmarchicus*) were purchased from Brine Shrimp Direct (sales@brineshrimpdirect.com; accessed on 4 April 2022). *Acartia tonsa* (*A. tonsa*) and *Labidocerca aestiva* (*L. aestiva*) were both harvested via netting from the Gulf of Mexico in May of 2022. Sea water was filtered in a plankton net with a 200 μm mesh, and the copepods were identified and separated under 400× magnification light microscopy. Copepods were washed in distilled water and frozen at −20 °C for future analysis. Microalgae (*Isochrysis galbana*) were obtained from Algae Research & Supply (Carlsbad, CA, USA) for comparison to copepods, since microalgae are a major source of lipids and lipid precursors for copepods. This is only one of many species of microalgae consumed by copepods.

### 2.2. Lipidomics

The copepod and algal samples (*n* = 5) were sonicated in 2 mL of methanol:water (1:1) containing stable isotope internal standards. These included 2 nmoles [^2^H_5_]DHA and 2 nmoles [^13^C_3_]diacylglycerol 36:2. Next, 2 mL of methyl-tert-butyl ether were added prior to vigorous shaking at room temperature for 30 min. To attain phase separation, the samples were centrifugated at 4000× *g* for 30 min. at room temp. One mL of the upper organic layer was isolated and dried by centrifugal vacuum evaporation. The lower layer was dried at 80 °C overnight, and the resulting dried pellets weighed to obtain the dry weight for each sample.

The dried organic extracts were dissolved in infusion solution (2-propanol: methanol: chloroform: H_2_O (160:80:80:1) with the water containing 15 mg of NH_4_Cl) for mass spectrometric analyses. For flow infusion analysis (FIA), the samples were infused at 12 μL per min into the ESI source for high-resolution data acquisition (140,000, <2 ppm mass error), with an orbitrap mass spectrometer (Thermo Q Exactive). The specific adducts monitored are listed in data Tables [12,46,47]. Between injections, the transfer line was washed with successive 800 µL washes of methanol and hexane/ethyl acetate/chloroform/H_2_O (3:2:1:0.1). Structural validation was achieved via tandem mass analyses (MS^2^). Product ions were monitored with <2 ppm mass error. For MS^2^ analyses, an isolation window of 0.4 amu and collision energies of 15, 25, and 35 NCE (arbitrary units) were used.

### 2.3. Data Analysis

HR-MS provides high quality data with <2 ppm mass error, and in the majority of cases <1 ppm. Within a given lipid family, this allows for clear separation of family members with similar but not identical masses. In the case of isobars, lipids with the same exact mass but different chemical structures, tandem mass spectrometry (MS^2^) often allows differentiation, since product ions are collected with high resolution. In cases where HR-MS is insufficient to resolve compounds, chromatographic methods prior to HR-MS are required.

We have built an Excel spreadsheet database of over 12,000 unique lipids which includes exact masses and calculated ion adducts. Based on our infusion solvent, the predominant ions were [M+H]^+^, [M-H_2_O+H]^+^, or [M+NH_4_]^+^ in positive electrospray ionization (PESI), while they were [M-H]^−^ or [M+Cl]^−^ in NESI. For lipids forming a chloride adduct, the Cl^37^ isotope was also monitored to validate the lipid identity. The HR-MS scan data were imported into this Excel spreadsheet, and any mass with less than 2 ppm mass error for the calculated ion adduct was recorded as a hit; the peak intensity for each hit was imported into the spreadsheet if greater than 100,000 integrated counts (S/N > 3). The exact masses used to build our database were obtained (December 2022) from LipidMaps (lipidmaps.org), Human Metabolome Database (https//hmdb.ca), Mycobacterium Lipid Database (mrl.colostate.edu/mtb), Chemspider (https://www.chemspider.com), Seaweed Metabolite Database (SWMD), and PubChem (https://pubchem.ncbi.nlm.nih.gov). Since these databases are not all inclusive or always up-to-date with regard to invertebrate lipids, we also extensively used scientific publications to expand the database. This is essential for copepod and microalgal lipids.

For relative quantitation, the peak intensities of individual lipids were divided by the peak intensity of the appropriate internal standard. The internal standard for negative ESI (NESI; 2 nmoles [^2^H_5_]DHA) or for positive ESI (PESI; 2 nmoles [^13^C_3_]diacylglycerol 36:2) were used in these calculations. The values obtained were corrected for the sample dry weight, which ranged from 5 to 20 mg, and are presented as mean ± standard deviation (SD) in Tables and as a summary Figure with bar plots created in R Version 4.2.3 (R Core Team, 2023, R: A language and environment for statistical computing. R Foundation for Statistical Computing, Vienna, Austria. URL https://www.R-project.org/), to visualize lipid ratio trends across copepod species. 

For MS^2^ studies, parent ions were selected with a 0.4 amu window and product ions monitored with 140,000 resolution (<2 ppm mass error). The HCD energies used were 15, 25, and 35 arbitrary units [46,47].

### 2.4. Nomenclature

Lipid nomenclature adheres to the guidelines of Lipid Maps [48]. Carbon positions are counted from the terminal carboxy group. Lipid notation such as 20:5 indicates 20 carbons and 5 double bonds. Lyso is shorthand for loss of a fatty acid substitution.

## 3. Results and Discussion

### 3.1. Overview

The following section integrates the background, our findings, and relevance of these observations to a planned long-term prospective study of the consequences of climate change on the lipidome of oceanic copepods.

### 3.2. Carotenoid Pigments

Carotenoids are organic pigments synthesized from lipid precursors by algae, bacteria, fungi, and plants [49]. The long hydrocarbon chains of these pigments span membranes with the terminal ring groups residing at the interior and exterior surfaces of membranes. These terminal ring groups act as antioxidants at the membrane surfaces. Copepods utilize carotenoids, which they ingest with phytoplankton [50], as protective pigments against the oxidative stress of short-wavelength solar radiation [51,52].

In copepods and microalgae (*Isochrysis galabana*), we found high levels of the carotenoids phoenicoxanthin and astaxanthin, along with fatty acyl esters of astaxanthin in copepods (Table 1). These included myristyl (14:0), behenyl (22:0), and cervonyl (22:6) esters, which have previously been reported for krill, another crustacean [53,54]. Red carotenoid pigments are generated by metabolism of yellow dietary precursors from microalgae [38,40]. Dietary pigments include 3-hydroxyechinenone, canthaxanthin, zeaxanthin, adonirubin, and adonixanthin. We did not observe any of these carotenoids or apocarotenoids [55] in copepods. We therefore speculate that dietary pigments are rapidly converted to astaxanthin and phoenicoxanthin by mitochondrial metabolism in copepods [42]. Therefore, at this time, we anticipate that astaxanthin levels in copepods may have the potential to be biomarkers of the health of the microalgal community that provides a food reservoir to copepods, which are filter feeders, and of copepod mitochondrial function. These future data will supply valuable information regarding the interplay of these oceanic species.

Carotenoid identities were validated by MS^2^ of the [M+H]^+^ cations. In all cases, the product ions were monitored with less than 1 ppm mass error for [Toluene–H_2_]^+^ = [91.0542]^+^ and [MH-Toluene]^+^ = [MH 92.0526]^+^. While these data validate that the monitored lipids were carotenoids, it cannot distinguish between potential isobaric carotenoids.

### 3.3. Copepodamides

Copepodamides are polar isoprenoid fatty acids with a terminal amide linkage to the amino acid taurine. These amino lipids are produced and released into the water solely by copepods, where they stimulate toxin production by phytoplankton to evade predation [40,56,57]. Copepodamides (=CH_2_ at C2) and dihydrocopepodamides (-CH_3_ at C2) possess a fatty acid acyl linkage at C3 [40,58].

In our study we only monitored copepodamides in *C. finmarchicus* (Table 2). High levels of the lyso forms were measured, along with lower levels of the palmitoyl (16:0) and gaidyl (16:1) acyl esters. The lack of copepodamides in *A. tonsa* and *L. aestiva* may relate to the Gulf of Mexico environment, which possesses a much richer food source for copepods. It is more essential to synthesize copepodamides to protect less-rich food sources from predation by other species [56]. The high levels of lysocopepodamide measured in *C. finmarchicus* suggest that this may be a storage pool of this precursor of copepodamides that are synthesized for secretion.

Copepodamide structures were validated by MS^2^ of the [M-H]^−^ anions (Supplementary File). In all cases, the product ions were monitored with less than 1 ppm mass error for the terminal taurine [124.0074]^−^ and for the base scaffold [C_22_H_41_NO_5_S]^−^ = [430.2633]^−^, with loss of the acyl substitutions at position C5.

Since copepodamides are lipids that are unique to copepods, monitoring this lipid family will provide biomarkers of *C. finmarchicus* viability and ability to participate in the maintenance of their major food source, marine phytoplankton.

### 3.4. Wax Esters and Triacylglycerols

The two major lipid families that serve as energy reservoirs are triacylglycerols (TG) and wax esters (WE). In the case of copepods, wax esters are generally stored at higher concentrations [32,35,36,59,60,61]. WE are generated by acylation of a fatty alcohol [62]. First, fatty-acyl-CoA reductase (FAR; EC 1.2.1.84) converts a fatty acyl-CoA to the corresponding fatty aldehyde, which in turn is converted to the fatty alcohol by fatty aldehyde reductase (FALR; EC 1.1.1.2). Acylation of the fatty alcohol involves acyl-CoA wax alcohol acyltransferase (AWAT1; EC 2.3.1.75), which has been reported for algae [63] but requires confirmation in copepods. Wax ester metabolism occurs via wax ester hydrolase (WEH; EC 3.1.1.50), which has been characterized in plants but not in copepods at this time.

Our analyses demonstrated high levels of wax esters in copepods and microalgae (*Isochrysis galabana*) (Table 3). The higher molecular weight wax esters and triacylglycerols possessed significant levels of the fatty acids 20:4, 20:5, 22:5, and 22:6 (Table 3), suggesting that these lipids also function as storage pools of this critical polyunsaturated fatty acid (PUFA). *C. finmarchicus* was unique in that wax esters were the dominant lipid storage pool. This may be the result of the cold-water environment of *C. finmarchicus.* Previous work has demonstrated that cold-water copepods like *Tigriopus kingsejongensis* have greater lipid metabolism than Pacific Ocean copepods like *Tigriopus japonicus* [64].

TGs were not detected in *C. finmarchicus*, which had the highest levels of WE (Table 3). WE serve as a rich energy resource pool in the copepods *C. finmarchicus, Calanus helgolanicus,* and *Gaussia princeps* [34,65]. In contrast, in the hunter copepod species *L. aestiva*, TGs predominated, presumably a biomarker of the more complex diet of this species. As with WEs, TGs also were found to have PUFA constituents (Table 3).

### 3.5. Monoacylglycerols (MG) and Modified MGs

Monoacylglycerols (MG) have diverse functions that include storage pools of fatty acids, membrane structure, and signal transduction. We monitored MGs with saturated fatty acid and PUFA substituents in copepods and microalgae (*Isochrysis galabana*) (Table 4).

We also report, for the first time, alanyl-MGs (Table 4) in copepods and microalga (*Isochrysis galabana*). This finding was validated via monitoring the product cation of 90.0550 for alanine in MS^2^ experiments. Alanyl-phosphatidylglycerols (Ala-PG) and alanyl-phosphatidyl-ethanolamines (Ala-PE) have been reported for many Gram-positive bacterial strains, but not alanyl-MGs [66,67,68]. These aminoacylations modify the membrane charge in bacteria. It remains to be determined if the alanyl-MGs we monitored are synthesized by copepods or their gut bacteria, or if they are degradation products of Ala-PG and Ala-PE. However, we did not monitor Ala-PGs or Ala-PEs in our analysis.

There are also a number of betaine lipids that have been reported for bacteria, fungi, and microalgae [69,70]. Monoacylglyceryl trimethylhomoserine (MGTS) is one of these betaine lipids which we monitored in copepods and microalga. Structural validation was obtained with the product cation of 104.1075 for choline in MS^2^ experiments (Supplementary File), however, monoacylglyceryl hydroxymethyltrimethyl-β-alanine (MGTA) is a structural isomer of MGTS that we cannot rule out at this time. It is important to note that all previous publications of algae have monitored only MGTS and not MGTA. While MGTSs may be acquired via the diet, the biosynthetic pathway for these lipids may well be present in copepods. This involves the initial formation of an aminolipid by the reaction of S-adenosylmethionine with either an MG or a DG. Next, the homoserine addition to the glycerol backbone is sequentially methylated to generate MGTSs. In the green alga *Chlamydomonas reinhardtii*, a single DGTS synthase protein (BTA1Cr) co-ordinates all of these reactions [71].

MGTSs, like phosphatidylcholines (PC), are quaternary amines, making them zwitterionic membrane lipids. However, they are connected to the glycerol backbone via an ether linkage, which is more flexible than the phosphodiester linkage of a PC, more chemically stable, and more stable against degradation by phospholipases, presumably improving membrane stability in an aquatic environment [71].

The third betaine lipid family in algae and plants is monoacylglyceryl carboxyhydroxy-methylcholine (MGCC) which is present in the non-plasmid membrane fraction where it also can substitute for PCs [72]. We monitored, and report for the first time, high levels of MGCCs in C. finmarchicus (Table 4). MGCC structures were validated via identification of the product cation of 104.1070 for choline in MS^2^ experiments. It remains to be determined if these MGCCs are dietary or synthesized by copepods.

### 3.6. Diacylglycerols (DG) and Modified DGs

We monitored high levels of DGs in copepods but much lower levels in *Isochrysis galabana* (Table 5). DGs are essential for membrane function both as structural lipids and as precursors for structural GPLs. DGs are also important lipids in the membranes of the nuclear envelope, endoplasmic reticulum [73], and in the Golgi for transport carrier biogenesis [74]. DGs are involved in signal transduction pathways via activation of protein kinases and by direct modulation of nuclear signal transduction [75].

As with the MGs, we also monitored the trimethylhomoserine modification of DGs (DGTS) in copepods and microalga (*Isochrysis galabana*) (Table 5). DGTSs have been reported for microalgae [64,69,70,71], but this is the first report of these lipids in copepods. As with MGTSs, structural validation for DGTSs was obtained with the product cation of 104.1075 for choline in MS^2^ experiments. However, DGTAs are possible isobars. In microalgae, DGTSs substitute for glycerophospholipids in membranes, thereby conserving phosphate but also function as a source for triacylglycerols under stress conditions, thereby maintaining a major energy source [76].

We also monitored the carboxyhydroxymethylcholine modification of MGs (MGCC) and DGs (DGCC) in copepods and microalgae (*Isochrysis galabana*) (Table 5). DGCCs which were detected at higher levels than MGCCs in the microalga *I. galabana* and the copepods *A. tonsa* and *L. aestiva* (Table 5), suggesting that MGCCs may be deacylation products (i.e., lyso) of DGCCs. *C. finmarchicus* lacked detectable DGCCs.

Major saccharolipids that are integral to photosynthetic membranes are mono- and di-galactosyl DGs (MGDG and DGDG, respectfully). In the case of microalga (*Isochrysis galabana*), we monitored both MGDGs and DGDGs (Table 5). In this regard, previous studies have demonstrated that MGDG levels in microalgae are decreased with exposure to ocean acidification [20]. As expected, these lipids were not monitored in copepods since they lack chloroplasts.

### 3.7. Docosahexaenoic Acid (DHA)

Copepods have the enzyme machinery [34] required for the biosynthesis of docosahexaenoic acid (DHA; FA 22:6n-3). Our data (Table 6) validate that copepods maintain very high levels of DHA and hydroxy-DHA isoforms, oxylipin metabolites of DHA [77,78,79,80,81]. Oxylipins are also synthesized by closely related krill [80]. We monitored these oxylipins in copepods, where they may come from their diet but are more likely synthesized by the copepods. Our data cannot distinguish the many isoforms of hydroxy-DHA. The complex roles of oxylipins in cellular regulation in copepods require further study. In contrast to copepods, low levels of DHA and hydroxy DHA were monitored in microalga (*Isochrysis galabana*).

### 3.8. Sterols: Cholesterol

Cholesterol is the most abundant sterol in the membranes of copepods [82,83,84]. This sterol is critical for the formation of a liquid-ordered phase in membranes, thereby providing membrane stabilization/fluidity and maintenance/restriction of membrane permeability across a range of body temperatures [84]. Cholesterol is also contained within lipid rafts, regulating the integration of receptor, enzyme, transporter, and ion channel proteins. In our samples, we, like previous researchers [83], observed that copepods have high levels of cholesterol and cholesterol esters (Table 6). It should be cautioned that, without chromatography, our cholesterol measurements may also include other sterol isobars at the same monitored mass; however, chromatography of copepod lipid extracts support the identity of cholesterol [83]. We also report, for the first time, that microalgae (*Isochrysis galabana*) have high levels of galactosyl/glucosyl cholesterol.

In contrast to copepods, hopanoids are the major membrane sterols in eubacteria, but not archaebacteria. Both sterols and hopanoids are polycyclic triterpene products of the metabolic precursor squalene. Archaebacteria are conjectured to utilize polyterpenes (polyprenols, carotenoids, and quinones) as membrane regulators [84].

### 3.9. Glycerophospholipids (GPL)

The chemical skeleton of glycerophospholipids (GPL) includes a glycerol back bone with one (i.e., Lyso-GPL) or two (i.e., diacyl, also termed phosphatidyl) fatty acid substituents and a polar phosphodiester head group. In the case of this study, we detected GPL with headgroups of phosphocholine, phosphoethanolamine, and phosphoglycerol.

Copepods were found to contain choline and ethanolamine GPL with the rank order of *C. finmarchicus* >> *L. aestiva* > *A. tonsa* (Table 7). In the cases of PCs and PEs, PUFAs were present in a large number of these GPLs (Table 7). This profile for PCs is similar to that previously reported for krill [85]. No phosphatidylcholines were detected in the microalgae (*Isochrysis galabana*), along with low levels of phosphatidylethanolamines. However, phosphatidylglycerols were only monitored in the microalgae (*Isochrysis galabana*) (Table 7) and did not contain PUFAs. Previous work has demonstrated that ocean acidification results in elevated levels of both phosphatidylglycerols and phosphatidylethanolamines in microalgae [86]. These data demonstrate the sensitivity of lipidomics analyses in monitoring the effects of climate change on marine organisms.

PCs were the predominant GPL in all three copepod species (Table 7), and relative levels were higher than what we monitored with DGTSs (Table 5), lipid substitutes for PCs.

PC and PE are isobaric with, for example, PC 36:0 = PE 39:0. Since PCs do not form [M-H]^−^ ions, reliable measurement of PEs in negative ESI is possible. We also performed MS^2^ analyses of the observed PEs and monitored the expected product [PE headgroup]^−^ = [140.0118]^−^. In positive ESI, the PEs could contribute to the PC signals; however, this contribution would come from odd-carbon PCs, which are rare. In addition, we performed MS^2^ analyses of the PCs and monitored the expected product [Phosphocholine]^+^ = [184.0739]^+^.

Since our analytical platform accurately quantitates both PCs and DGTSs, calculating a ratio of the levels of PCs to DGTSs could be a future useful index of phosphorous under- or over-supply with environmental stressors, based on prior studies of fungi [43], bacteria [44], and algae [86,87].

### 3.10. Sphingolipids: Ceramides

The basic skeleton of a ceramide is a sphingolipid base with an amide-linked fatty acid. For all but one class of sphingolipids, the sphingolipid base is sphingosine or sphinganine, both generated by addition of a long-chain CoA to the carboxy function of serine. In contrast, in the case of deoxyceramides, the addition of the long-chain CoA is to alanine, which lacks a hydroxy group, hence they are termed deoxyceramides. In the biosynthesis of both ceramides and deoxyceramides, a second fatty acid undergoes N-acylation to the amine group of serine or alanine, respectfully. With the absence of the 1-hydroxyl group in the sphingolipid base, deoxyceramides cannot be converted into ceramide phosphates, hexosylceramides, or sphingomyelins (SM). In the case of *C. finmarchicus*, which had the highest levels of deoxyceramides, this copepod species had the smallest variety of SM variants (Table 8).

The loss of the hydroxy function in deoxyceramides results in a significant reduction in the ability of deoxyceramides to hydrogen bond, making them more hydrophobic [88,89]. Ceramides function as structural components of membranes and in signal transduction pathways. In this regard, a number of studies indicate that ceramides [89] and deoxyceramides [89] are activators of autophagy and mitophagy. Specifically, deoxyceramides augment the autophagic flux via inducing the accumulation of phagosomes and lysosomes, thereby regulating cellular proliferation/differentiation and programmed cell death.

High levels of deoxyceramides were detected in copepods but not microalgae (*Isochrysis galabana*), with the rank order of *C. finmarchicus* >>> *L. aestiva* ≈ *A. tonsa* (Table 8). This is the first report of these sphingolipids in copepods. The identity of deoxyceramides was validated by monitoring the dehydrated deoxybases in MS^2^ experiments. These bases included m18:2 (264.2686) for deoxyceramides 34:2 and 36:2, m18:3 (262.2529) for deoxyceramides 34:3 and 36:3, and m18:4 (260.2373) for deoxyceramides 34:4 and 36:4.

The functions of deoxyceramides in copepods remain to be defined, but the greater lipophilicity of these unique lipids may be important for their roles in membrane function in species with lower GPL content. In the study of climate change effects on microalgae, sphingolipid levels (ceramides) were augmented [90].

### 3.11. Sphingolipids: Sphingomyelins

Copepods and closely related krill possess significant levels of sphingomyelins (SM) [91,92,93]. This is a result of the unique feature of these invertebrates possessing myelinated axons [92,93]. In contrast to mammals, the dominant sphingomyelin was not SM 42:5;O2. We monitored a variety of sphingomyelins, with each copepod species possessing a unique fingerprint for these sphingolipids (Table 8). No hydroxy-sphingomyelins were detected in copepods and no sphingomyelins were detected in microalgae (*Isochrysis galabana*). Since SM are isobaric with ceramide phosphoethanolamines [94], we performed MS^2^ analyses and monitored the expected SM product ion [Phosphocholine]^+^ = [184.0739]^+^ with < 1 ppm mass error. Monitoring SMs in future studies may be useful, since these lipids represent a nervous system biomarker.

### 3.12. Copepod Heterofibrins

Lipid droplets are cellular organelles that store cholesterol, cholesterol esters, and acylglycerols (mainly TGs). These organelles are critical in energy metabolism via regulation of the cellular levels of free lipids. This involves a complex integration of lipid storage, hydrolysis, and transport [95]. The levels of lipid droplets have been shown to vary with the reproductive cycle in *C. finmarchicus* [96]. Heterofibrins have been described as small molecule modulators of lipid droplet formation [96]. Heterofibrins encompass two families of 18 and 19 carbon fatty acids possessing a unique diyne-ene moiety. Analogs within each family have a carboxy-terminal monolactyl or dilactyl substitution [96]. These complex lipids were initially isolated from *Spongia (Heterofibria)*. Ours is the first data to demonstrate the presence of heterofibrins in the copepod *C. finmarchicus* (Table 9). We monitored both heterofibrin A2 (HF-A2) and heterofibrin B2 (HF-B2); the structures were validated by MS^2^ of the [M-H]^−^ anions (Supplementary File). Both heterofibrins generated product ions for lactic acid [89.0244]^−^ and propionic acid [73.0294]^−^ of the carboxy terminal. Both heterofibrins also had product ions that resulted from cleavage at the diyne-ene moiety closest to the carboxy terminal, resulting in product ions of [173.1336]^−^ for HF-A2 and [187.1492]^−^ for HFB2. All product ions were monitored with less than 1 ppm mass error.

These data support a complex regulation of lipid droplet storage in cold-water copepods. Hence, heterofibrins may prove to be useful as biomarkers of the complex effects of climate change on energy metabolism in *C. finmarchicus.*

### 3.13. Microalgal Chlorophylls

Microalgae are a large photosynthetic biomass that represents a significant feedstock for many aquatic inhabitants [90]. In our analysis of *I. galabana*, we demonstrated that pheophytin a [97] is the major chlorophyll in this microalgae (Table 9), which are known to utilize pheophytin a and chlorophyll a for photosynthesis [97]. In contrast, chlorophylls were not detected in copepods, which do not utilize photosynthesis as an energy source. These data also suggest that any dietary chlorophylls are presumably rapidly digested after ingestion.

### 3.14. Bacillariolides

Bacillariolides are heterocyclic (hydroxycyclopentafuranone) oxylipins with a 13 carbon isopentyl sidechain that have only previously been reported in diatoms [98]. This is the first report of these oxylipins in copepods, with none being detected in algae (*Isochrysis galabana*). Bacillariolide II and its methoxymethyl derivative were monitored in all three copepod species (Table 10). Presumably, the 13-carbon isoprenoid chain inserts into membranes and the terminal cyclopentafuranone ring contributes to membrane charge.

Product ions of the [M-H]^−^ molecular ions were fragments of the lipid sidechain (Supplementary File). These included [C_10_H_16_]^−^ = [135.1179]^−^ and [C_12_H_18_]^−^ = [161.1336]^−^, all monitored with less than 1 ppm mass error. The function(s) of these novel marine oxylipins remains to be determined.

## 4. Summary

**Figure 1:**
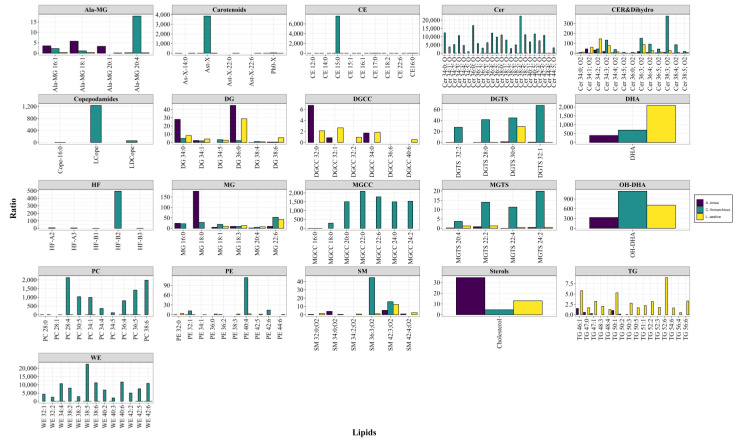
Relative levels (R) of lipids in copepods. Levels are presented (mean values) as a ratio of the peak intensity of each lipid to the peak intensity of the internal standard (2 nanomoles [^2^H_5_]DHA) corrected for the sample dry weight. (*n* = 5). Ala, alanine; ASt-X, astaxanthin; CE, cholesterol ester; Cer, ceramide; Cope, copepodamide; DG, diacylglycerol; DGCC, diacylglyceryl carboxyhydroxymethylcholine; DGTS, diacylglyceryl trimethylhomoserine; DHA, FA 22:6; HF, heterofibrin; L, lyso; MG, monoacylglycerol; OH-DHA, hydroxy FA 22:6; PC, phosphatidylcholine; PE, phosphatidylethanolamine; SM, sphingomyelin; TG, triacylglycerol; WE, wax ester.

Our lipidomics analytical platform, with the desired characteristics of high precision and accuracy, will allow for quantitation of lipid adaptations to the stressors of climate change. In fact, lipidomics has already been shown to quantify the effects of herbicide exposure, as an experimental stressor, on microalgae [99] and lipid changes in heat-stressed mussels [100]. The lipidomics platform we have established provides the technology required to monitor copepod species-specific lipid adaptations to climate change as well as shifts in their food sources [101]. This is essential for the quantification of long-term multigenerational responses of this oceanic sentinel species to altering climate conditions. As presented in Figure 1, the lipidome of each copepod species is very unique, and our data clearly indicate that detailed lipidomics analyses will be required to evaluate the effects of climate stressors on the lipidome of each copepod species.

## 5. Conclusions

While lipid biochemistry and nomenclature are extremely complex, it is not essential for all readers interested in climate change to understand all of the various lipid classes presented in this study. The clear message from this research is that we can monitor key copepod lipid families with high accuracy and, therefore, potentially monitor lipid families that respond to environmental perturbations evoked by climate change. Ultimately, it is the goal of this research to identify sensitive lipid biomarkers of climate change.

### Study Limitations

Biological: Copepods have complex life cycles and their lipidomes will vary with those cycles and the seasons. We have only taken a snapshot of one point in time with this probe study. However, these analyses have established the major lipid families to monitor in our longitudinal studies. Larger sample sets from different seasons of the year will be crucial to build a lipid database for a long-term climate change study.

Technical: Our HR-MS analytical platform (<2 ppm mass error), that utilizes both PESI and NESI, significantly reduces the risk of lipid misassignments. However, there are a number of lipid structural isobars that require MS^2^ and/or TLC evaluations for full structural validation. Over the last 10 years, our Metabolomics Unit has built a database of a number of these specific issues and optimal technical solutions. Specific issues include our inability to distinguish between: (i) a cyclopropyl group and a double bond in a fatty acid chain, and (ii) an added methyl group vs. addition of a CH_2_ in a fatty acid chain. Again, MS^2^ and/or TLC evaluations will be our first strategies with lipids of high interest. NMR may be considered if required, but this involves significant scale-up and purification methods due to the low sensitivity of NMR.

## Figures and Tables

**Table 1:** Relative levels of carotenoids and carotenoid fatty esters in copepods and microalga. Levels are presented as a ratio of the peak intensity of each lipid to the peak intensity of the internal standard (2 nanomoles [^13^C_3_]triacylglycerol 48:0), corrected for the sample dry weight. Results are presented as Mean ± SD (*n* = 5). -, not detected.

Carotenoids	[M+H]^+^	*Isochrysis galbana*	*Acartia tonsa*	*Calanus finmarchicus*	*Labidocerca aestiva*
Phoenicoxanthin	581.3989	566.9 ± 124.8	3.34 ± 1.23	32.8 ± 15.3	1.04 ± 0.72
Astaxanthin	597.3938	18.56 ± 4.32	2.46 ± 1.07	3854.0 ± 200.8	16.5 ± 7.0
Astaxanthin-14:0	807.5922	-	0.99 ± 0.12	-	10.2 ± 3.6
Astaxanthin-22:0	919.7174	-	-	-	2.34 ± 1.34
Astaxanthin-22:6	907.6235	-	-	-	3.63 ± 0.66

**Table 2:** Relative levels of copepodamides in *C. finmarchicus*. Levels are presented as a ratio of the peak intensity of each lipid to the peak intensity of the internal standard (2 nanomoles [^2^H_5_]DHA), corrected for the sample dry weight. Results are presented as Mean ± SD (*n* = 5).

Copepodamides	[M-H]^−^	*Calanus finmarchicus*
Lysocopepodamide	448.2739	1240 ± 329
Lysodihydrocopepodamide	450.2895	59 ± 13
Copepodamide-16:0	686.5035	2.11 ± 1.20

**Table 3:** Relative levels of wax esters (WE) and triacylglycerols (TG) in copepods and microalga. Levels are presented as a ratio of the peak intensity of each lipid to the peak intensity of the internal standard (2 nanomoles [^13^C_3_]triacylglycerol 48:0), corrected for the sample dry weight. Results are presented as Mean ± SD (*n* = 5). -, not detected.

Wax Esters	[M+NH_4_]^+^	*Isochrysis galbana*	*Acartia tonsa*	*Calanus finmarchicus*	*Labidocerca aestiva*
WE 32:1	496.5088	-	-	4318 ± 2414	-
WE 32:2	494.4932	-	-	2516 ± 1392	0.058 ± 0.017
WE 34:4	518.4932	-	0.54 ± 0.25	10,686 ± 5960	1.50 ± 0.28
WE 38:2	578.5871	18.35 ± 4.84	-	8009 ± 4530	-
WE 38:3	576.5714	2.67 ± 0.77	0.091 ± 0.008	2835 ± 1618	-
WE 38:5	572.5401	2.41 ± 1.23	0.13 ± 0.019	22,497 ± 12,673	0.16 ± 0.017
WE 38:6	570.5245	-	-	11,219 ± 6374	0.018 ± 0.006
WE 40:2	606.6184	0.20 ± 0.026	-	6865 ± 3916	-
WE 40:3	604.6027	0.16 ± 0.024	0.71 ± 0.062	2042 ± 1423	-
WE 40:6	598.5558	1.21 ± 0.034	-	11,687 ± 6611	-
WE 42:2	634.6497	0.31 ± 0.051	-	5028 ± 2764	-
WE 42:5	628.6027	0.038 ± 0.008	4.63 ± 2.25	7584 ± 4321	2.64 ± 0.065
WE 42:6	626.5871	1.20 ± 0.23	1.08 ± 0.23	10,882 ± 6056	0.66 ± 0.18
**Triacylglycerols**	**[M+NH_4_]^+^**	***Isochrysis galbana***	***Acartia tonsa***	***Calanus finmarchicus***	***Labidocerca aestiva***
TG 46:1	780.7076	-	1.51 ± 0.75	-	5.81 ± 3.00
TG 47:0	810.7545	-	0.57 ± 0.26	-	1.70 ± 0.88
TG 47:1	808.7389	-	0.29 ± 0.15	-	3.24 ± 1.57
TG 48:3	818.7232	-	-	-	2.01 ± 1.00
TG 48:4	816.7076	-	-	-	1.28 ± 0.65
TG 50:1	850.7858	-	1.02 ± 0.17	-	5.29 ± 2.89
TG 50:2	848.7702	-	0.15 ± 0.05	-	-
TG 50:3	846.7545	-	0.042 ± 0.021	-	2.79 ± 1.45
TG 50:5	842.7232	-	-	-	1.68 ± 1.02
TG 51:1	864.8015	-	-	-	2.20 ± 1.01
TG 52:2	876.8015	-	-	-	3.19 ± 1.66
TG 52:3	874.7858	-	-	-	1.75 ± 0.090
TG 52:6	868.7389	-	-	-	8.97 ± 3.12
TG 54:6	896.7702	-	-	-	1.63 ± 0.83
TG 56:4	928.8328	-	-	-	0.51 ± 0.15
TG 56:6	924.8015	-	-	-	3.31 ± 1.53

**Table 4:** Relative levels of monoacylglycerols (MG) and modified MGs in copepods and microalga. Levels are presented as a ratio of the peak intensity of each lipid to the peak intensity of the internal standard (2 nanomoles [^2^H_5_]DHA for NESI and 2 nanomoles [^13^C_3_]triacylglycerol 48:0 for PESI), corrected for the sample dry weight (Mean ± SD; *n* = 5). -, not detected.

Monoacylglycerols	[M+Cl]^−^	*Isochrysis galbana*	*Acartia tonsa*	*Calanus finmarchicus*	*Labidocerca aestiva*
MG 16:0	365.2469	1.76 ± 0.094	24.09 ± 14.64	21.70 ± 3.08	-
MG 18:0	393.2782	0.73 ± 0.079	177.7 ± 88.2	28.43 ± 6.91	-
MG 18:1	391.2625	3.31 ± 0.061	5.07 ± 0.79	19.07 ± 8.00	10.11 ± 4.78
MG 18:3	387.2312	0.29 ± 0.011	9.35 ± 4.64	9.42 ± 1.95	13.26 ± 4.20
MG 20:4	413.2469	1.20 ± 0.13	1.59 ± 0.58	5.45 ± 1.49	7.28 ± 3.73
MG 22:6	437.2469	1.32 ± 0.10	9.26 ± 0.26	52.88 ± 12.05	42.74 ± 20.38
**Alanyl-monoacyl-glycerols**	**[M+H]^+^**	***Isochrysis galbana***	***Acartia tonsa***	***Calanus finmarchicus***	***Labidocerca aestiva***
Ala-MG 16:1	400.3057	0.20 ± 0.063	3.55 ± 0.57	2.24 ± 0.81	0.34 ± 0.012
Ala-MG 18:1	428.3370	0.27 ± 0.084	5.77 ± 0.35	1.14 ± 0.26	0.30 ± 0.11
Ala-MG 20:1	456.3683	0.029 ± 0.013	3.31 ± 1.72	-	0.13 ± 0.070
Ala-MG 20:4	450.3214	-	0.20 ± 0.11	17.8 ± 7.10	0.18 ± 0.051
**MGTS**	**[M+H]^+^**	***Isochrysis galbana***	***Acartia tonsa***	***Calanus finmarchicus***	***Labidocerca aestiva***
MGTS 20:4	522.3789	-	0.19 ± 0.028	3.74 ± 2.56	1.26 ± 0.58
MGTS 22:2	554.4415	1.77 ± 0.71	0.86 ± 0.51	14.01 ± 4.07	1.29 ± 0.85
MGTS 22:4	550.4102	-	0.044 ± 0.021	11.37 ± 3.32	0.52 ± 0.20
MGTS 24:2	582.4728	0.83 ± 0.38	0.48 ± 0.21	19.88 ± 5.12	0.59 ± 0.20
**MGCC**	**[M+H]^+^**	***Isochrysis galbana***	***Acartia tonsa***	***Calanus finmarchicus***	***Labidocerca aestiva***
MGCC 16:0	490.3738	-	0.95 ± 0.03	0.22 ± 0.23	0.83 ± 0.40
MGCC 18:0	518.4051	-	-	293.0 ± 69.6	-
MGCC 20:0	546.4364	-	-	1504 ± 1002	-
MGCC 22:0	574.4677	-	-	2100 ± 66	-
MGCC 22:6	562.3738	0.18 ± 0.12	-	1781 ± 798	-
MGCC 24:0	602.4990	-	-	1498 ± 516	-
MGCC 24:2	598.4677	-	-	1534 ± 424	-

Ala, Alanine; MGCC, Monoacylglyceryl carboxyhydroxymethylcholine; MGTS, Monoacylglyceryl trimethylhomoserine.

**Table 5:** Relative levels of diacylglycerols (DG) and modified DGs in copepods and microalga. Levels are presented as a ratio of the peak intensity of each lipid to the peak intensity of the internal standard (2 nanomoles [^2^H_5_]DHA) for NESI and 2 nanomoles [^13^C_3_]triacylglycerol 48:0 for PESI), corrected for the sample dry weight. Results are presented as Mean ± SD (*n* = 5). -, not detected.

Diacylglycerols	[M+Cl]^−^	*Isochrysis galbana*	*Acartia tonsa*	*Calanus finmarchicus*	*Labidocerca aestiva*
DG 34:0	631.5078	0.12 ± 0.022	28.1 ± 14.6	4.94 ± 1.35	8.26 ± 4.40
DG 34:1	629.4922	1.14 ± 0.14	2.45 ± 1.24	1.99 ± 0.20	4.25 ± 1.29
DG 34:5	621.4296	0.18 ± 0.039	-	3.38 ± 0.92	2.81 ± 0.64
DG 36:0	659.5391	-	45.1 ± 5.24	2.37 ± 1.01	28.8 ± 10.1
DG 38:4	679.5078	0.012 ± 0.005	0.022 ± 004	1.19 ± 0.46	0.78 ± 0.44
DG 38:6	675.4765	0.37 ± 0.04	0.54 ± 0.10	0.52 ± 0.11	5.81 ± 2.12
**DGTS**	**[M+H]^+^**	***Isochrysis galbana***	***Acartia tonsa***	***Calanus finmarchicus***	***Labidocerca aestiva***
DGTS 28:0	656.5460	1.99 ± 0.64	0.019 ± 0.009	41.6 ± 22.5	0.16 ± 0.025
DGTS 30:0	684.5773	0.67 ± 0.23	1.29 ± 0.67	44.8 ± 21.9	029 ± 0.18
DGTS 32:1	710.5929	39.7 ± 5.8	0.63 ± 0.075	67.6 ± 23.7	0.069 ± 0.033
DGTS 32:2	708.5773	1.97 ± 0.37	0.024 ± 0.004	27.7 ± 6.2	-
**DGCC**	**[M+H]^+^**	***Isochrysis galbana***	***Acartia tonsa***	***Calanus finmarchicus***	***Labidocerca aestiva***
DGCC 32:0	728.6035	0.81 ± 0.045	6.74 ± 2.12	-	2.12 ± 1.2
DGCC 32:1	726.5878	4.23 ± 0.94	0.86 ± 0.51	-	2.66 ± 1.37
DGCC 32:2	724.5722	2.18 ± 1.0	-	-	0.93 ± 0.13
DGCC 34:0	756.6348	-	1.72 ± 0.91	-	1.84 ± 1.00
DGCC 36:6	772.5722	5.41 ± 1.05	-	-	-
DGCC 40:6	828.6348	0.18 ± 0.051	-	-	0.51 ± 0.11
**Monogalactosyl DG**	**[M+Cl]^−^**	***Isochrysis galbana***	***Acartia tonsa***	***Calanus finmarchicus***	***Labidocerca aestiva***
MGDG 32:1	763.5137	12.31 ± 3.91	-	-	-
MGDG 32:2	761.4981	4.07 ± 1.12	-	-	-
MGDG 32:3	759.4824	2.04 ± 0.65	-	-	-
MGDG 32:4	757.4668	3.90 ± 1.26	-	-	-
MGDG 32:5	755.4511	1.22 ± 0.42	-	-	-
MGDG 34:1	791.5450	4.25 ± 1.29	-	-	-
MGDG 34:2	789.5294	3.96 ± 1.16	-	-	-
MGDG 34:6	781.4668	1.57 ± 0.48	-	-	-
MGDG 36:2	817.5607	11.13 ± 3.40	-	-	-
MGDG 36:3	815.5450	1.92 ± 0.51	-	-	-
MGDG 36:4	813.5294	1.39 ± 0.39	-	-	-
MGDG 36:5	811.5137	2.55 ± 0.86	-	-	-
MGDG 36:6	809.4981	2.64 ± 0.76	-	-	-
**Digalactosyl DG**	**[M+Cl]^−^**	***Isochrysis galbana***	***Acartia tonsa***	***Calanus finmarchicus***	***Labidocerca aestiva***
DGDG 30:1	897.5352	1.11 ± 0.60	-	-	-
DGDG 30:2	895.5196	0.49 ± 0.16	-	-	-
DGDG 32:1	925.5665	0.51 ± 0.04	-	-	-
DGDG 32:4	919.5196	0.33 ± 0.01	-	-	-
DGDG 34:3	949.5665	0.22 ± 0.11	-	-	-
DGDG 34:5	945.5396	2.79 ± 1.25	-	-	-
DGDG 36:3	977.5978	3.96 ± 1.16	-	-	-
DGDG 36:4	975.5822	0.64 ± 0.17	-	-	-
DGDG 36:5	973.5665	0.42 ± 0.19	-	-	-
DGDG 36:6	971.5509	0.22 ± 0.05	-	-	-

DGCC, Diacylglyceryl carboxyhydroxymethylcholine; DGTS, Diacylglyceryl trimethylhomoserine.

**Table 6:** Relative levels of docosahexaenoic acid (DHA; PUFA 22:6), cholesterol, and cholesterol esters (CE) in copepods and microalga. Levels are presented as a ratio of the peak intensity of each lipid to the peak intensity of the internal standard (2 nanomoles [^2^H_5_]DHA for NESI and 2 nanomoles [^13^C_3_]triacylglycerol 48:0 for PESI), corrected for the sample dry weight. Results are presented as Mean ± SD (*n* = 5). -, not detected.

PUFA	[M-H]^−^	*Isochrysis galbana*	*Acartia tonsa*	*Calanus finmarchicus*	*Labidocerca aestiva*
DHA	327.2330	0.57 ± 0.055	394 ± 153	696 ± 149	2090 ± 769
Hydroxy-DHA	343.2279	0.36 ± 0.10	333 ± 67	1139 ± 278	712 ± 310
**Cholesterol**	**[MH-H_2_O]^+^**	***Isochrysis galbana***	***Acartia tonsa***	***Calanus finmarchicus***	***Labidocerca aestiva***
Cholesterol	369.3516	-	34.58 ± 15.34	4.59 ± 2.12	12.98 ± 2.43
**Cholesterol Esters**	**[M+NH_4_]^+^**	***Isochrysis galbana***	***Acartia tonsa***	***Calanus finmarchicus***	***Labidocerca aestiva***
Hexosyl-Cholesterol *	566.4415	591 ± 252	-	-	-
CE 12:0	586.5558	-	0.66 ± 0.13	-	0.11 ± 0.04
CE 14:0	614.5871	-	4.11 ± 1.83	-	1.51 ± 0.74
CE 15:0	628.6027	-	4.63 ± 2.24	7584 ± 4521	2.64 ± 0.66
CE 15:1	626.5871	-	1.08 ± 0.40	-	0.66 ± 0.18
CE16:0	642.6184	-	1.76 ± 0.89	12.44 ± 4.75	0.90 ± 0.22
CE 16:1	640.6027	-	4.31 ± 0.23	-	3.63 ± 0.69
CE 17:0	656.6340	-	0.30± 0.99	20.70 ± 1.78	1.03 ± 0.26
CE 18:2	668.6340	-	4.94 ± 1.26	-	2.90 ± 0.10
CE 22:6	714.6184	-	2.28 ± 0.93	-	1.71 ± 0.84

*, hexosyl = glucosyl and/or galactosyl.

**Table 7:** Relative levels of glycerophospholipids in copepods and microalga. Levels are presented as a ratio of the peak intensity of each lipid to the peak intensity of the internal standard (2 nanomoles [^2^H_5_]DHA) for NESI and 2 nanomoles [^13^C_3_]triacylglycerol 48:0 for PESI), corrected for the sample dry weight. Results are presented as Mean ± SD (*n* = 5). -, not detected.

Phosphatidylcholine (PC)	[M+H]^+^	*Isochrysis galbana*	*Acartia tonsa*	*Calanus finmarchicus*	*Labidocerca aestiva*
PC 28:0 (14:0/14:0)	678.5068	-	3.44 ± 1.23	-	5.91 ± 2.34
PC 28:1	676.4912	-	-	-	0.39 ± 0.21
PC 28:4	670.4442	-	-	2134 ± 658	3.03 ± 0.75
PC 30:5	696.4599	-	-	1037 ± 324	-
PC 34:1 (16:0/18:1)	760.5851	-	0.92 ± 0.21	995 ± 473	2.24 ± 0.90
PC 34:4	754.5381	-	-	360 ± 114	-
PC 34:5	752.5225	-	-	116 ± 56	-
PC 36:4	782.5694	-	0.73 ± 0.11	806 ± 52	1.60 ± 0.87
PC 36:5	780.5538	-	2.70 ± 0.34	1416 ± 745	1.37 ± 0.29
PC 38:6 (18:1/20:5)	806.5694	-	-	1984 ± 868	-
**Phosphatidylethanolamine (PE)**	**[M-H]^−^**	***Isochrysis galbana***	***Acartia tonsa***	***Calanus finmarchicus***	***Labidocerca aestiva***
PE 32:0 (16:0/16:0; 18:0/14:0)	690.5079	-	0.35 ± 0.05	-	4.27 ± 2.1
PE 32:1	688.4923	0.14 ± 0.04	0.19 ± 0.06	11.70 ± 1.25	0.29 ± 0.12
PE 34:1	716.5236	0.025 ± 0.011	-	-	0.17 ± 0.08
PE 36:0	746.5705	-	-	-	1.94 ± 0.93
PE 36:2	742.5392	0.051 ± 0.020	0.68 ± 0.21	-	-
PE 38:3	768.5549	-	-	-	2.07 ± 0.88
PE 40:4	794.5702	-	-	114 ± 51	2.59 ± 0.11
PE 42:5	820.5862	-	-	-	1.69 ± 0.79
PE 42:6 (20:0/22:6)	818.5705	-	-	13.99 ± 3.81	-
PE 44:6	846.6018	-	-	-	0.67 ± 0.33
**Phosphatidylglycerol (PG)**	**[M-H]^−^**	***Isochrysis galbana***	***Acartia tonsa***	***Calanus finmarchicus***	***Labidocerca aestiva***
PG 30:0 (15:0/15:0)	693.4712	1.23 ± 0.62	-	-	-
PG 32:1	719.4869	0.63 ± 0.23	-	-	-
PG 34:1 (16:0/18:1)	747.5182	3.51 ± 1.35	-	-	-
PG 34:2	745.5025	2.51 ± 0.99	-	-	-
PG 36:2 (18:1/18:1)	773.5338	7.58 ± 3.06	-	-	-
PG 36:3	771.5182	1.27 ± 0.51	-	-	-

**Table 8:** Relative levels of sphingolipids (ceramides and sphingomyelins) in copepods and microalga. Levels are presented as a ratio of the peak intensity of each lipid to the peak intensity of the internal standard (2 nanomoles [^2^H_5_]DHA for NESI and 2 nanomoles [^13^C_3_]triacylglycerol 48:0 for PESI), corrected for the sample dry weight. Results are presented as Mean ± SD (*n* = 5). -, not detected.

Deoxyceramides	[M+H]^+^	*Isochrysis galbana*	*Acartia tonsa*	*Calanus finmarchicus*	*Labidocerca aestiva*
Cer 34:0; O	524.5401	-	-	12,363 ± 6942	-
Cer 34:1; O	522.5245	-	-	3871 ± 2188	-
Cer 34:2; O	520.5088	-	-	5258 ± 2970	0.42 ± 0.064
Cer 34:3; O	518.4932	-	-	10,686 ± 5960	1.50 ± 0.28
Cer 34:4; O	516.4775	-	-	4711 ± 1458	0.70 ± 0.17
Cer 34:5; O	514.4619	-	-	978 ± 562	-
Cer 36:0; O	552.5714	-	-	16,760 ± 9450	-
Cer 36:1; O	550.5558	-	-	5883 ± 3285	-
Cer 36:2; O	548.5401	-	0.14 ± 0.04	3146 ± 1805	0.11 ± 0.05
Cer 36:3; O	546.5245	-	0.11 ± 0.06	6435 ± 3759	0.25 ± 0.13
Cer 36:4; O	544.5088	-	0.61 ± 0.31	12,199 ± 6893	0.76 ± 0.02
Cer 36:5; O	542.4932	-	0.058 ± 0.023	9800 ± 5450	0.11 ± 0.02
Cer 38:0; O	580.6027	-	-	11,112 ± 6221	-
Cer 38:1; O	578.5871	-	-	8009 ± 4500	-
Cer 38:2; O	576.5714	-	0.091 ± 0.008	2835 ± 1618	-
Cer 38:3; O	574.5558	-	-	5059 ± 2750	0.079 ± 0.039
Cer 38:4; O	572.5401	-	0.13 ± 0.01	22,497 ± 12,676	0.16 ± 0.01
Cer 38:5; O	570.5245	-	-	11,220 ± 6341	0.018 ± 0.006
Cer 40:1; O	606.6184	-	-	6865 ± 3716	-
Cer 40:5; O	598.5558	-	-	11,687 ± 6211	-
Cer 42:4; O	628.6027	-	4.63 ± 2.25	7584 ± 4012	2.64 ± 0.66
Cer 42:5; O	626.5871	-	1.08 ± 0.22	10,822 ± 6156	0.66 ± 0.18
Cer 44:4; O	656.6340	-	0.21 ± 0.11	20.7 ± 1.7	1.03 ± 0.26
Cer 44:5; O	654.6184	-	0.094 ± 0.01	3289 ± 1642	0.18 ± 0.04
**Ceramides**	**[M+Cl]^−^**	***Isochrysis galbana***	***Acartia tonsa***	***Calanus finmarchicus***	***Labidocerca aestiva***
Cer 34:0; O2	574.49758	0.69 ± 0.09	2.33 ± 0.10	8.17 ± 3.2	12.16 ± 6.31
Cer 34:1; O2	572.48193	0.25 ± 0.09	43.45 ± 5.56	0.86 ± 0.23	60.3 ± 23.0
Cer 34:2; O2	570.46628	8.93 ± 0.28	32.91 ± 15.17	43.8 ± 10.4	145 ± 43
Cer 34:3; O2	568.45063	-	17.50 ± 6.56	132.4 ± 29.0	77.9 ± 19.7
Cer 34:4; O2	566.43498	-	4.75 ± 1.22	37.5 ± 7.6	13.7 ± 6.63
Cer 34:5; O2	564.41933	-	1.22 ± 0.34	9.00 ± 1.31	1.38 ± 0.52
Cer 36:0; O2	602.52888	1.93 ± 0.37	0.63 ± 0.24	8.38 ± 2.56	2.67 ± 1.89
Cer 36:3; O2	596.48193	-	17.25 ± 6.59	151 ± 30	86.8 ± 28.0
Cer 36:4; O2	594.46628	-	7.18 ± 3.77	91.5 ± 20.5	26.03 ± 14.63
Cer 36:5; O2	592.45063	-	3.44 ± 1.56	42.5 ± 8.4	11.69 ± 4.96
Cer 38:3; O2	568.45063	-	8.28 ± 1.86	375 ± 86	27.03 ± 8.33
Cer 38:4; O2	566.43498	-	1.80 ± 0.34	85.9 ± 22.5	6.63 ± 2.21
Cer 38:5; O2	564.41933	-	2.12 ± 0.45	18.6 ± 3.0	6.72 ± 3.23
Cer 40:4; O2	622.49758	-	3.98 ± 1.45	68.5 ± 16.4	6.62 ± 3.78
Cer 42:3; O2	652.54453	-	4.00 ± 1.10	421 ± 104	326 ± 54
Cer 42:4; O2	650.52888	-	18.81 ± 5.84	771 ± 210	66.3 ± 4.1
Cer 42:5; O2	648.51323	-	0.92 ± 0.26	24.2 ± 6.1	2.26 ± 1.0
Cer 44:3; O2	680.57583	-	16.76 ± 8.49	12.9 ± 3.7	130 ± 2
Cer 44:4; O2	678.56018	-	6.32 ± 2.30	274 ± 72	44.30 ± 1.05
Cer 44:5; O2	676.54453	-	-	9.77 ± 2.04	0.45 ± 0.17
Cer 46:4; O2	706.59148	-	-	19.5 ± 5.7	0.16 ± 0.09
**Sphingomyelins (SM)**	**[M+H]^+^**	***Isochrysis galbana***	***Acartia tonsa***	***Calanus finmarchicus***	***Labidocerca aestiva***
SM 32:0; O2	677.5592	-	0.42 ± 0.17	-	1.55 ± 0.38
SM 34:0; O2	705.5905	-	4.04 ± 2.03	-	0.44 ± 0.06
SM 34:2; O2	701.5592	-	-	-	0.81 ± 0.37
SM 36:3; O2	727.5749	-	-	44.83 ± 21.90	1.23 ± 0.60
SM 42:3; O2	811.6688	-	5.31 ± 1.89	15.76 ± 5.39	12.35 ± 5.62
SM 42:4; O2	809.6531	-	0.96 ± 0.48	-	2.51 ± 0.79
SM d18:1/25:3	823.6688	-	2.87 ± 1.21	-	20.21 ± 10.39
SM d18:1/25:4	821.6531	-	0.47 ± 0.14	-	1.45 ± 0.72
SM d18:1/26:2	839.7001	-	-	-	5.72 ± 2.39
SM d18:1/26:3	837.6844	-	0.40 ± 0.15	19.04 ± 8.32	1.46 ± 0.47

**Table 9:** Relative levels of heterofibrins in *C. finmarchicus* and chlorophylls in *I. galabana*. Levels are presented as a ratio of the peak intensity of each lipid to the peak intensity of the internal standard (2 nanomoles [^2^H_5_]DHA for NESI and 2 nanomoles [^13^C_3_]triacylglycerol 48:0 for PESI), corrected for the sample dry weight. Results are presented as Mean ± SD (*n* = 5).

Heterofibrins (HF)	[M-H]^−^	*Calanus finmarchicus*
HF-A2	345.2071	6.40 ± 1.33
HF-A3	417.2283	4.50 ± 1.54
HF-B1	287.2017	0.82 ± 0.15
HF-B2	359.2228	495 ± 105
HF-B3	431.2439	1.10 ± 0.58
**Chlorophylls**	**[M+H]^+^**	***Isochrysis galbana***
Chlorophyll a	911.5532	0.40 ± 0.12
Pheophytin a	872.5765	12.6 ± 3.7

**Table 10:** Relative levels of bacillariolides in *copepods*. Levels are presented as a ratio of the peak intensity of each lipid to the peak intensity of the internal standard (2 nanomoles [^2^H_5_]DHA) corrected for the sample dry weight. Results are presented as Mean ± SD (*n* = 5). -, not detected.

Bacillariolides	[M-H]^−^	*Isochrysis galbana*	*Acartia tonsa*	*Calanus finmarchicus*	*Labidocerca aestiva*
Bacillariolide II	315.1966	-	10.7 ± 4.8	27.6 ± 6.2	10.7 ± 3.1
Methoxymethyl-Bacillariolide II	359.2228	-	70.4 ± 38.4	494.8 ± 105.1	120.8 ± 60.2

## Data Availability

All data is included in the manuscript and Supplementary Materials.

## Supplementary Materials

The following supporting information can be downloaded at: https://www.mdpi.com/article/10.3390/life13122335/s1, Supplementary Figure S1. The MS^2^ product spectra for novel lipid observations.
