# Supplementary material for: Pilot Lipidomics Study of Copepods: Investigation of Potential Lipid-Based Biomarkers for the Early Detection and Quantification of the Biological Effects of Climate Change on the Oceanic Food Chain
Source: Life (Basel). 2023 Dec 13;13(12):2335. doi: 10.3390/life13122335 (PMC10744631; doi:10.3390/life13122335)
Supplement: Supplementary file 1 [file life-13-02335-s001.zip › life-2746561-supplementary.pdf]

Supplementary File

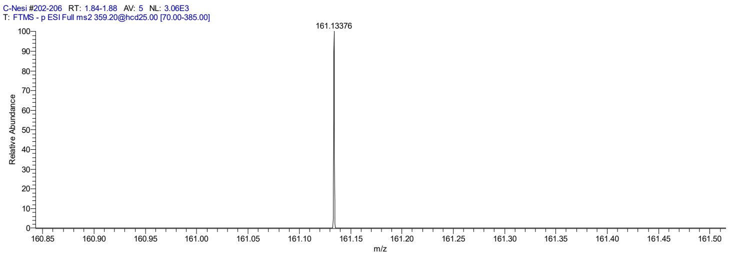

A. MS[2] product of bacillariolide II (359.2) extracted from *C. finmarchicus*. $[C_{12}H_{18}]^-$ = 161.1337 ((0.62 ppm).

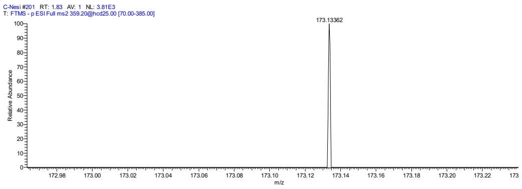

B. MS[2] product of heterofibrin A2 (345.2) extracted from *C. finmarchicus*. [diyne-ene moiety closest to the carboxy terminal]$^-$ = 173.13362 (0.12 ppm).

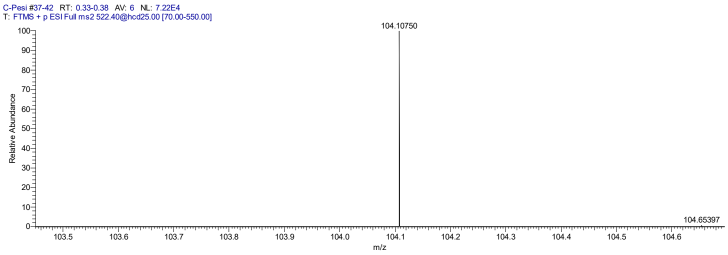

C. MS[2] product of MGTS 20:4 (522.4) extracted from *C. finmarchicus*. [choline]$^+$ = 104.1075 (0.29 ppm).

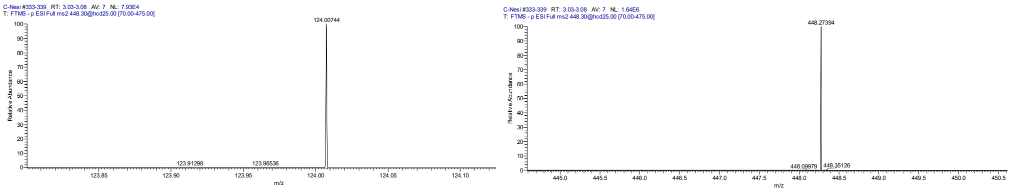

D. MS[2] product of lysocopepodamide (448.3) extracted from *C. finmarchicus*. [taurine]$^-$ = 124.074 (0.32 ppm) and [base scaffold of $C_{22}H_{41}NO_5S + H_2O]^-$ = 448.2739 (0.18 ppm).

Supplementary Figure S1. The MS[2] product spectra for novel lipid observations.
